# The impact of different time intervals between hCG priming and oocyte retrieval on ART outcomes

**Published:** 2013-07

**Authors:** Fatemeh Ghasemian, Roya Faraji, Maryam Asgharnia, Ziba Zahiri, Mohammad Hadi Bahadori

**Affiliations:** 1 *Biology Faculty, Kharazmi University, Tehran, Iran.*; 2 *Reproductive Health Research Center, Alzahra Educational and Remedial Center, Guilan University of Medical Sciences, Rasht, Iran. *; 3 *Cellular and Molecular Research Center, Faculty of Medicine, Guilan University of Medical Sciences, Rasht, Iran.*

**Keywords:** *Oocyte retrieval time*, *Human Chorionic Gonadotropin priming time*, *Oocyte morphology*, *Embryo quality*, *Assisted reproductive technique*

## Abstract

**Background:** Abnormal oocyte morphology has been associated with the hormonal environment to which the gametes are exposed.

**Objective:** In this study, we evaluated the oocytes morphology, fertilization rate, embryos quality, and implantation rate resulted of retrieved oocytes in different times after human chorionic gonadotrophin (HCG) administration.

**Materials and Methods:** A total of 985 metaphase II oocytes were retrieved 35, 36, 37 and 38 h after the injection of HCG as groups 1, 2, 3, and 4 respectively. Oocyte morphology was divided into (I) normal morphology, (II) extracytoplasmic abnormalities, (III) cytoplasmic abnormalities and (IV) intracytoplasmic vacuoles and in each group, oocytes were evaluated according to this classification.

**Results:** Extracytoplasmic abnormalities were encountered in 17.76% and 31.1% of these oocytes (groups 3 and 4 respectively, p=0.007) in comparison with 12.23% group 2. Cytoplasmic abnormalities in group 4 were higher than other groups. 23.88% (p=0.039) and 43.25% (p=0.089) of resulted 2PN (two pronucleus) from groups 3 and 4 showed grade Z3 respectively in comparison to group 2 (16.44%). Normal and various categories of abnormal oocytes did not differ regarding fertilization and cleavage rates (p=0.061). However, group 4 showed significant difference in the rate of embryos fragmentation (grade III and IV embryo) in comparison with group 2 (40.96% vs. 24.93%, p=0.078). The pregnancy rate was higher in G2 and G3 groups (28.5 and 24.13% respectively).

**Conclusion:** Oocyte retrieval time following HCG priming affected on oocyte morphology, 2PN pattern and embryos qualities subsequently. Both good quality embryo formation and pregnancy outcomes were noticeably higher when oocytes were retrieved 36 h after HCG priming in ART program.

## Introduction

Natural endogenous luteinizing hormone (LH) surge does not appear, or has appeared with improper timing and magnitude in woman who undergo controlled ovarian hyperstimulation (COH) during assisted reproduction technique (ART). Therefore, exogenouse LH, human chorionic gonadotropin (HCG), was commonly used. HCG similar to LH triggers the final follicular maturation before oocyte retrieval (1). 

Many substance influences ART results by affect follicular development, oocyte maturation, fertilization and embryo development subsequently. The effect of these factors depends on HCG priming time (2-4). Therefore, quality of oocyte and embryo was influenced by these factors (1). After the onset of LH surge, range of ovulation time and average time are from 24 to 56 h and 32 h respectively (5). 

In most ART centers, commonly practiced interval was done 32-36 h after HCG priming in patients that ovulation induction was stimulated by using Clomiphene Citrate (CC) and/or human menopausal gonadotropin (HMG). Highly expanded cumulus of retrieved oocytes increased the prolonged luteinization, which may reflect oocyte maturation (1). Therefore, fertilization and cleavage rate of these oocytes also increased, indicating that a longer interval may result in gamete quality by allowing more optimal in vivo maturation. 

Wang *et al* reported that oocyte maturation rate was higher in the long interval group (>36 h) compared with short interval group (<36 h) (1). Also, they reported that there was no significant difference between the two groups with regard to fertilization, implantation and pregnancy rates. 

On the other hand, abnormal oocyte morphology has been associated with stimulation the hormonal environment to which the gametes are exposed and chromosomal constitution of the oocyte itself (2). De Sutter *et al* reported that there were no relationship between the oocyte morphology and fertilization after intracytoplasmic sperm injection (ICSI) (3). In other study, Serhal *et al* showed that embryos derived from morphologically abnormal oocytes result in poor pregnancy rates (4).

Little attention has been focused on oocyte morphology obtained in different times of oocyte retrieval after HCG priming. In this way, this study was evaluated the influence of the oocyte retrieval time (35, 36, 37, and 38 h) after HCG priming on oocytes and embryo morphology, fertilization rate, and pregnancy outcome with a standard ovarian stimulation protocol. 

## Materials and methods

**Patients**

This analytic research was approved in accordance with the guidelines of the Guilan University of Medical Science (GUMS) Ethical Committee and performed in the infertility therapy center of Alzahra Hospital. Informed consent was obtained from all patients. The study material consisted of 985 metaphase II (MII) oocytes obtained from 126 ICSI cycles undertaken for male infertility. 

Patient selection was based on the following criteria: Patient age: 26-32 years; sperm concentration; <20×10^6^/mL; sperm morphology: >20%; motility: >40%; progression: >20%, and leukocyte: 0-4×10^6^/mL. The data collection period was from September 2010 to May 2011. Only cycles stimulated with a long gonadotrophin-releasing hormone (GnRH) analogue regimen combined with pure follicle-stimulating hormone (FSH, e.g. Gonal F; Merck, Switzerland) and with human menopausal gonadotropin (HMG, e.g. Merional, Menogon; Fering, Germany) were included in the study. 

Because of unexpected problems, oocyte retrieval was performed 35, 36, 37, and 38 h after the injection of HCG (e.g. Pregnyl; Daroupakhsh, Iran) that this study was considered as groups 1, 2, 3, and 4 respectively. Human chorionic gonadotrophin was administrated as a single dose of 10000 IU when the leading follicle reached 20 mm in average diameter in the presence of at least two other follicles of >18 mm in size. 

**Morphology assessment**

Approximately 2-4 h after oocyte retrieval, and the following incubation of oocytes in 80 IU/ml hyaluronidase for <30s, cumulus-corona cells were stripped off the oocytes with gentle pipetting. The morphology of the oocyte was assessed under an inverted microscope (IX70, Olympus, Japan) at ×200 or ×400 magnification and the oocyte were grouped as follows: (I) normal oocytes, (II) oocytes with extracytoplasmic abnormalities (large perivitelline space and dark zona), (III) oocytes with cytoplasmic abnormalities (dark cytoplasm, granular cytoplasm and aggregates of smooth endoplasmic reticulum) and (IV) intracytoplasmic vacuoles.

After preparing the semen sample the ICSI procedure was performed following conventional techniques (6). Normal fertilization was assumed for oocytes which formed two pronuclei and two polar bodies. The best embryos assessed morphologically were transferred transcervically day 3 after oocyte retrieval. The zygote and embryo morphology were considered on the following grading criteria:

Z1 zygotes had equal numbers of nucleoli aligned at the pronuclear junction. The absolute number was not counted but was between three and seven. Z2 had equal numbers of nucleoli of equal sizes in the same nuclei but with one nucleus having alignment at the pronuclear junction and the other with scattered nucleoli. Z3 zygotes had equal numbers and sizes of nucleoli (between three and seven) which were equally scattered in the two nuclei. 

Z4 zygotes were those with pronuclei that were not aligned, were of grossly different sizes or were not located in the central part of the zygote (7). The embryos were graded morphologically according to Staessen *et al* (8). Grade I: even and homogenous blastomeres without fragmentation; Grade II: even and homogenous blastomeres with <20% fragmentation; Grade III: uneven and non-homogenous blastomeres with 20-50% fragmentation; Grade IV: uneven and non-homogenous blastomeres with >50% fragmentation.

Embryo transfer was performed using the Labotect catheter (Labotect, Germany). Transfers were performed with transvaginal ultrasound guidance. To support the luteal phase, 100 mg of progesterone i.m. (Gestone, Paines and Byrne, Greenford, Middlesex, UK) was administrated daily for 16 days. A urinary pregnancy test was performed 16 days after embryo transfer and when positive the first ultrasound scan was scheduled for 4 weeks after embryo transfer. The fertilization rate, embryonic quality and clinical pregnancy rates were determined in relation to oocyte morphology obtained in 35, 36, 37, and 38 h after HCG administration. 

**Statistical** **analysis**

Analysis was done using the SPSS 16.0 statistical package. All outcomes were assessed using Chi-squared test and student's *t-*test. A level of 0.05 or less was considered significant.

## Results

A total of 1082 oocytes were collected. Of these, 985 (91.43%) were in metaphase II and the remainder in metaphase I or germinal vesicle stage. The maturation rate was higher in G4 compared to other groups (94%, p=0.041). Oocyte morphology was considered in different groups (Table I) [groups G1- G4 respectly]. Normal oocytes were observed in 47.36% (n=20), 64.06% (n=45), 52.89% (n=38) and 25.83% (n=23) of retrieved oocytes after 35, 36, 37 and 38 h of HCG priming. 

There was significant difference between G2-3 and G1, 4 (p=0.037). Abnormalities of extra-cytoplasmic (large perivitellin space and dark zona) were seen in 31.1% of retrieved oocytes after 38h of HCG priming that this was significantly higher in comparison to other groups (p=0.007). Cytoplasmic abnormalities (dark cytoplasmic, granular cytoplasm and aggregates of SER) were significantly difference in G4 in comparison to other groups (p=0.068). Evidence of table I indicated that intracytoplasmic vacuoles were seen in 10.52% of oocytes for G4 in comparison to other groups (p=0.041). 

The rate of grade Z3 and Z4 zygotes was higher in G4. Furthermore, embryos derived from normal and abnormal oocytes were morphologically similar in G2 and G3 (p=0.059) (Table II). The mean number of embryo transferred was not different among groups. The pregnancy rate (with + β-HCG) was better in G2 and G3 (p=0.039). However, the results indicated the similar rate in implanted embryos among groups (p=0.063) (Table III). These groups did not differ from each other regarding mean male and female age, the mean number of retrieve oocytes, infertility duration, the number of gonadotropin ampoules injected and ovarian stimulation during.

**Table I:** Different time of oocyte retrieval after HCG priming and oocyte morphology

**Groups**	**G1 (n=20)**		**G2 (n=45)**		**G3 (n=38)**		**G4 (n=23)**	
	**n**	**Rate (%)**	**n**	**Rate (%)**	**n**	**Rate (%)**	**n**	**Rate (%)**
Retrieved metaphase II oocytes (% maturation)	133	(89.24)	384	(90.46)	259	(92.04)	209	(94^a^)
Normal oocyte	63	47.36	246^a^	64.06	137^a^	52.89	54	25.83
Extracytoplasmic abnormalities	26	19.54	47	12.23	46	17.76	65	31.1^b^
Cytoplasmic abnormalities	33	24.81	79	20.57	59	22.77	68	32.53^b^
Intracytoplasmic vacuoles	11	8.27	12	3.12	17	6.5	22	10.52^a^

**Group 1:** IVF cycles where oocytes were collected 35 h after HCG.

**Group 2:** IVF cycles where oocytes were collected 36 h after HCG.

**Group 3:** IVF cycles where oocytes were collected 37 h after HCG.

**Group 4:** IVF cycles where oocytes were collected 38 h after HCG.

Maturation rate (^a ^p=0.037).

Extracytoplasmic (^b^p=0.007).

Cytoplasmic (^b^p=0.068).

Intracytoplasmic vacuoles (^a^p=0.041).

Abnormalities were higher in G4 (p0.047).

Normal oocytes were higher in G2 and G3 (^a ^p=0.037). All outcomes were assessed using Chi-squared test.

**Table II:** Zygote and embryo quality derived from morphologically normal and abnormal oocytes

	**G1**		**G2**		**G3**		**G4**	
	**n**	**Rate (%)**	**n**	**Rate (%)**	**n**	**Rate (%)**	**n**	**Rate (%)**
Zygote	126		371		247		178	
Z1 2PN	24	19.04	148	39.89^b^	77	31.17^b^	16	8.98
Z2 2PN	50	39.68	135	36.38	85	34.41	66	37.07
Z3 2PN	36	28.57	61	16.44	59	23.88^a^	77	43.25^b^
Z4 2PN	16	12.69	27	7.27	26	10.52	19	10.67
Embryo	121	-	365	-	234	-	166	-
Grade I + II	82	67.76	274	75.06^b^	168	71.79^b^	98	59.03
Grade III+ IV	39	32.23	91	24.93	66	28.2	66	40.96^b^

**Group 1:** IVF cycles where oocytes were collected 35 h after HCG.

**Group 2:** IVF cycles where oocytes were collected 36 h after HCG.

**Group 3:** IVF cycles where oocytes were collected 37 h after HCG.

**Group 4:** IVF cycles where oocytes were collected 38 h after HCG. 2PN: Two pronucleus.

Zygote percentages with grade Z1 was higher in G2 and G3 (^a ^p=0.039, ^b ^p=0.067), and with grade Z3 was higher in G4 (^b^p=0.089). There was not significant difference in grades Z2 and Z4 among groups (p=0.059). Embryos with grade I+II were higher in G2 and G3 (^b^p=0.068) and grade III+IV were higher in G4 (^b^p=0.042). All outcomes were assessed using Chi-squared test.

**Table III:** Pregnancy and implantation rates after transfer embryo derived from morphologically normal and abnormal oocytes

	**G1 **	**G2 **	**G3 **	**G4 **
Mean no. of embryos transferred (n)	2.3	2.3	2.5	2.7
Pregnancy rate per transfer [n(%)]	5/24 (21.83)	14/49 (28.5)^a^	7/29 (24.13)	4/24 (16.66)
Implanted embryos/implantation rate	10.1	13.2	10.7	9.8

**Group 1:** IVF cycles where oocytes were collected 35 h after HCG.

**Group 2:** IVF cycles where oocytes were collected 36 h after HCG.

**Group 3:** IVF cycles where oocytes were collected 37 h after HCG.

**Group 4:** IVF cycles where oocytes were collected 38 h after HCG.

There was not significantly difference in transferred embryo number among groups (p>0.05) and also implantation rate (p>0.05). Pregnancy rate was significantly higher in G2 in comparison to other groups (^a ^p<0.05). All outcomes were assessed using *t-*test.

## Discussion

Our results clearly indicate that oocyte morphology influences timing period after HCG priming. Oocyte morphology include perivitellin space and zona pellucida (extra-cytoplasmic) and cytoplasmic quality (present or absent dark/granular cytoplasm and aggregates of SER) and present or absent of vacuole. So that, the most normal oocyte morphology was retrieved in 36 and 37 h after HCG priming. The normal oocyte morphology was observed in G2 (36 h after HCG priming) especially. However, better zygotes obtained in this group (grade Z1 and Z2). 

Therefore, resulted embryos were significantly with good quality in this group. Furthermore, the pregnancy rate was in G2 and G3 (28.5 and 24.13%) vs. 21.83 and 16.66% in G1 and G4 respectively. These findings confirmed the results of Thibault *et al* (9) that oocyte quality may be directly influenced by the hormonal environment, estrogen and progesterone (ovarian steroids) particularly. These hormones involved in the initiation of cytoplasmic maturation and the final stage of nuclear maturation of the oocytes. When oocytes were retrieved later, oocytes exposed ovarian steroids for longer time. The ideal LH activity, administered as HMG, rLH or HCG in ART procedures (10). 

In common practice, human chorionic gonadotrophin (HCG) is used as a substitute for the mid-cycle LH surge, due to the degree of homology between the two hormones (11). Though, follicular development up to the preantral stage infeasible in the absence of LH, an essential role for this gonadotrophin for antral formation as well as further growth and differentiation has been uniformly recognized. LH plays a key role in both oocyte and follicular cells development through modification of the steroid and protein micro- and macro-environment (10, 12, 13). These physiological changes have a prominent role in oocyte, maturation, the process of ovulation and subsequent fertilization and implantation (10). It is well known that follicular development is highly dependent on pituitary secretion of FSH and LH. 

These hormones are essential for normal follicular E_2 _production, an action presented in the literature as the two-cell two-gonadotrophin theory (10). Serum E_2_ levels were measured by Beretsos *et al* which indicated that higher serum E_2_ levels detected on the day of HCG administration (ovulation induction, 1643.5±800.2) vs. 259.3±175.8 on the day 5 of FSH administration. Serum E_2_ levels per follicle on the day of HCG administration also tended to be higher (10). Although ovarian stimulation with gonadotrophin has the obvious benefit of increasing the number of oocytes available for treatment, it can also compromise oocyte quality leading to chromosomal anomalities and interference with imprinting during pre-implantation development (11, 14).

Therefore, the performance of oocyte retrieval in the suitable time may be useful and effect on embryo quality and pregnancy rate subsequently. However, this is the first study to report the effect of the timing between HCG priming and oocyte retrieval on oocyte morphology, zygote and embryo quality, pregnancy rate, and implantation percentage subsequently in ART program. In notice to this, the time that oocytes expose hormonal environment can differ. Therefore, the effect of this environment can evaluate on oocyte morphology in detail. Wang *et al* did a meta-analysis about this effect in two groups, long interval group (>36 h) and short interval group (<36 h) in the ART treatment cycles (1). This study expressed that fertilization, implantation, and pregnancy rates could not increase in the prolonged interval group.

The HCG was administrated when a sufficient number of follicles had developed, typically more than three follicles ≥17 mm in mean diameter, and the transvaginal ultrasound (TUS) guided approach was used for oocyte retrieval. Since the hours after luteinizing stimulus is a period of intense nuclear and cytoplasmic activity in human oocytes, so the interval between HCG priming and oocyte retrieval probably determines the degree of cellular and cytogenetic maturation (1).

In this study, our concern was the relationship between the time retrieval of oocyte after HCG priming (35, 36, 37 and 38 h after HCG priming) and oocyte morphology. So that, longer time after HCG injection was increased PVS and granular cytoplasm in oocyte retrieved. These abnormalities caused low zygote and embryo quality after ICSI. Also, the pregnancy rate was influenced but the implantation rate did not differ. So that both good quality embryo formation and pregnancy outcomes were noticeably higher when oocytes were retrieved 36 h after HCG priming in ART program.
